# Differential gene expression in LPS/IFNγ activated microglia and macrophages: *in vitro* versus *in vivo*

**DOI:** 10.1111/j.1471-4159.2009.05984.x

**Published:** 2009-05

**Authors:** Christoph D Schmid, Benoit Melchior, Kokoechat Masek, Shweta S Puntambekar, Patria E Danielson, David D Lo, J Gregor Sutcliffe, Monica J Carson

**Affiliations:** * Swiss Institute of Bioinformatics, Epalinges s/LausanneSwitzerland; † Division of Biomedical Sciences, Center for Glial-Neuronal Interactions, University of California RiversideCalifornia, USA; ‡ Department of Molecular Biology, The Scripps Research InstituteLa Jolla, California, USA

**Keywords:** autoimmunity, Clast 1, Nasu-Hakola, neurodegeneration, neuroinflammation, Torid

## Abstract

Two different macrophage populations contribute to CNS neuroinflammation: CNS-resident microglia and CNS-infiltrating peripheral macrophages. Markers distinguishing these two populations in tissue sections have not been identified. Therefore, we compared gene expression between LPS (lipopolysaccharide)/interferon (IFN)γ-treated microglia from neonatal mixed glial cultures and similarly treated peritoneal macrophages. Fifteen molecules were identified by quantative PCR (qPCR) as being enriched from 2-fold to 250-fold in cultured neonatal microglia when compared with peritoneal macrophages. Only three of these molecules (C1qA, Trem2, and CXCL14) were found by qPCR to be also enriched in adult microglia isolated from LPS/IFNγ-injected CNS when compared with infiltrating peripheral macrophages from the same CNS. The discrepancy between the *in vitro* and *in vivo* qPCR data sets was primarily because of induced expression of the ‘microglial’ molecules (such as the tolerance associated transcript, Tmem176b) in CNS-infiltrating macrophages. Bioinformatic analysis of the ∼19000 mRNAs detected by TOGA gene profiling confirmed that LPS/IFNγ-activated microglia isolated from adult CNS displayed greater similarity in total gene expression to CNS-infiltrating macrophages than to microglia isolated from unmanipulated healthy adult CNS. *In situ* hybridization analysis revealed that nearly all microglia expressed high levels of C1qA, while subsets of microglia expressed Trem2 and CXCL14. Expression of C1qA and Trem2 was limited to microglia, while large numbers of GABA+ neurons expressed CXCL14. These data suggest that (i) CNS-resident microglia are heterogeneous and thus a universal microglia-specific marker may not exist; (ii) the CNS micro-environment plays significant roles in determining the phenotypes of both CNS-resident microglia and CNS-infiltrating macrophages; (iii) the CNS microenvironment may contribute to immune privilege by inducing macrophage expression of anti-inflammatory molecules.

The CNS is supported and defended by two different macrophage populations: CNS-resident microglia and CNS-infiltrating macrophages (reviewed in Carson *et al.* 2007). In histological sections, these two cell types cannot be reliably distinguished from one another because CNS-resident microglia express most (if not all) common macrophage markers. In addition, both cell types can acquire either stellate or amoeboid morphologies depending on the CNS micro-environment. Despite this high degree of similarity, a large number of functional studies using flow cytometry and irradiation bone marrow chimeric mouse methodologies have convincingly demonstrated that these cells are functionally distinct (Hickey and Kimura 1988; Sedgwick *et al.* 1991; Vallieres and Sawchenko 2003; Byram *et al.* 2004; and reviewed in Ransohoff 2007).

Most tissue macrophages are relatively short-lived and are continually being replenished from bone marrow-derived cells (reviewed in Carson *et al.* 2006a,b; and Ransohoff 2007). By contrast, irradiation bone marrow chimeric mice reveal that CNS-resident microglia are largely self-renewing and rarely replenished from the bone marrow. Recent studies using non-irradiated parabiotic mice have confirmed these observations (Ajami *et al.* 2007). Irradiation bone marrow chimeric mice have also been used to selectively express major histocompatability complex (MHC) class II in either the radiation-insensitive (microglia) or radiation-sensitive compartment (peripheral CNS-infiltrating immune cells). These studies demonstrated that while peripheral immune cells were highly effective at initiating pro-inflammatory lymphocyte responses, CNS-resident microglia were not (Hickey and Kimura 1988; Greter *et al.* 2005). Rather CNS-resident microglia appeared to play an opposing role either in limiting pro-inflammatory lymphocyte responses or in initiating neuroprotective CD4+ T-cell responses (Byram *et al.* 2004; Takahasi *et al.* 2007).

Although CNS-resident microglia cannot be distinguished histologically from other macrophage populations, they can be distinguished using flow cytometry. Specifically, in CNS cell suspensions, CNS-resident microglia can be distinguished from acutely infiltrating peripheral macrophages by their lower levels of CD45 expression. Although the two levels of CD45 expression do slightly overlap, the bulk of each population can be reliably distinguished by this method (Sedgwick *et al.* 1991; Renno *et al.* 1995; Carson *et al.* 1998, 2006a,b; Fig. 1a). The stable differences in CD45 expression levels are also likely to have functional consequences. CD45 (also known as leukocyte common antigen) is a protein tyrosine phosphatase that has been implicated as an inhibitory receptor for a CD22; a ligand expressed by both CNS neurons and B cells (Mott *et al.* 2004). These data suggest that CNS neurons should be more effective at inhibiting infiltrating macrophages than CNS-resident microglia even when both are found in the same inflammatory foci! Considered together, these data demonstrate the continuing need to define the molecular differences between CNS-resident microglia and CNS-infiltrating macrophages.

**Fig. 1:**
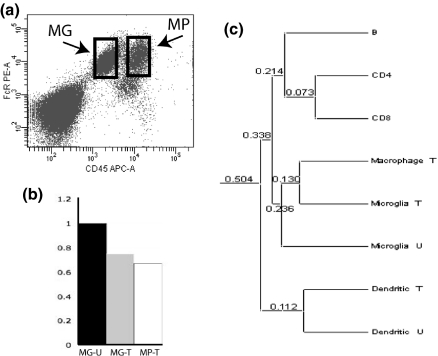
Microglia and macrophages isolated from the CNS show similar patterns of gene expression. (a): CNS-resident microglia (MG-boxed cells) and acutely infiltrating macrophages (MP-boxed cells) can be isolated from the same LPS/IFNγ-injected mouse brain and separated into two distinct cell populations using fluorescence-activated cell sorting. Microglia are defined as the Fc receptor positive, CD45lo cells (left box), and CNS-infiltrating macrophages are defined as the Fc receptor positive, CD45hi cells. (b) qPCR analysis of Tmem176b expression in microglia isolated from healthy unmanipulated adult CNS (MG-U), microglia isolated from LPS/IFNγ injected mouse CNS (MG-T) and acutely infiltrating macrophages isolated from the same injected CNS (MP-T). (c) Approximately 19000 different mRNAs were detected by TOGA gene expression profiling of lymphocytes (B cells, CD4+ and CD8+ T cells) isolated by flow cytometry from lymph nodes of naïve adult mice; unactivated CD45lo microglia from unmanipulated healthy adult mice, activated CD45lo microglia and CD45 high macrophages isolated by flow cytometry from LPS/IFNγ-injected adult murine; and *in vitro* unactivated and LPS-activated bone marrow-derived dendritic cells. Between each of the samples, pair-wise comparisons were made of the numbers of mRNAs expressed and the relative expression levels of the approximately total 19000 mRNAs detected by TOGA. From these comparisons, linear correlation coefficients were calculated in a pair-wise fashion for each of the samples. The difference index (numeric values listed at each branch in c) between each sample was calculated as 1 minus the correlation coefficient.

Using CD45 expression levels as a cell type specific maker, small numbers of microglia and CNS-infiltrating macrophages can be isolated from adult rodent CNS by flow cytometric cell sorting (Sedgwick *et al.* 1991; Renno *et al.* 1995; Carson *et al.* 1998). However, only a few hundred thousand cells per brain can be isolated by this method, which is insufficient for many functional and molecular assays (Carson *et al.* 1998, 2006a,b). We and others have therefore used primary microglia from neonatal mixed glial cultures and thioglycolate-elicted peritoneal macrophages as models for activated CNS-resident microglia and CNS-infiltrating macrophages, respectively (Carson *et al.* 1998, Papenfuss *et al.* 2007). Primary cultured microglia from mixed glial cultures have advantages over the common microglia cell lines as models for microglia *in vivo*. Primary cultured microglia are not oncogene immortalized and they are differentiated in the presence of astrocytes and oligodendrocytes. Thioglycolate-elicited peritoneal macrophages have advantages over splenic macrophages and oncogene-transformed macrophage cell lines in that they have been recently induced to infiltrate the peritoneum in response to pathogen recognition receptors. Here we test the predictive nature of these commonly used models to identify cell-type specific markers able to distinguish CNS-resident microglia from CNS-infiltrating macrophages.

## Materials and methods

### Microglia isolation from mixed glial cultures

Mixed glial cultures were prepared as previously described (Carson *et al.* 1998, 1999). Briefly, brains from postnatal day 1–3 C57Bl/6J mice were stripped of meninges, and the cortices mechanically dissociated, seeded into T-75 flasks, and maintained in Dulbecco’s modified Eagle’s medium, supplemented with 10% fetal bovine serum and insulin (5 μg/mL). After 3 weeks, mixed glial cultures were trypsinized and incubated as a single cell suspension in Dulbecco’s modified Eagle’s media without phenol red for 30 min at 37°C to allow for the re-expression of trypsinized surface markers. Microglia were purified to > 98% by flow cytometry using phycoerythrin-conjugated antibodies directed against FcR/CD16/CD32 (Pharmingen, San Diego, CA, USA). Cytoplasmic mRNA was prepared from isolated cells immediately after isolation as previously described (Schmid *et al.* 2002).

### Peritoneal macrophage and bone marrow-derived dendritic cell preparation

Peritoneal macrophages were prepared as previously described (Carson *et al.* 1998, 1999). Briefly, 3.0 mL of aged sterile thioglycolate broth were injected into the peritoneum of C57Bl/6J mice (Difco, Detroit, MI, USA). Mice were killed 3 days post-injection and peritoneal macrophages were harvested after killing by rinsing the peritoneal cavity with two 5 mL washes of OM5 media supplemented with 5.0 U/mL heparin (Sigma, St. Louis, MO, USA). Exudate cells were plated and allowed to adhere. Non-adherent cells were removed by rinsing the cultures. Greater than 90% of the remaining adherent cells were macrophages (CD11b+ cells). Bone marrow-derived dendritic cells were prepared by standard methods and purified by flow cytometry as described in Carson *et al.* (1999). Lymphocytes were isolated from lymph nodes of naïve mice by flow cytometry as previously described in Carson *et al.* 1999.

### Microglia and macrophage isolation from healthy and inflamed adult CNS

Microglia were isolated as previously described (Carson *et al.* 1999) from the CNS of healthy adult (8–12 weeks old) C57Bl/6J mice or from mice injected intracerebrally with LPS (100 ng/mL) and interferon (IFNγ, 10 U/mL) in a total volume of 5 μL. In brief, mice were killed by halothane inhalation, and the brains of the mice rapidly removed and mechanically dissociated. The cell suspension was separated on a discontinuous 1.03/1.088 Percoll gradient and microglia/macrophages were collected from the interface as well as from the 1.03 Percoll fraction. Microglia and CNS-infiltrating macrophages were purified by flow cytometry using Allophycocyanin-conjugated antibodies against pan-CD45 and phycoerythrin-conjugated antibodies against FcR/CD16/CD32 (Pharmingen). Activated microglia were identified as FcR-positive, and CD45lo/intermediate and macrophages identified as FcR-positive, CD45hi.

### RNA extraction and reverse transcription

RNA was extracted from isolated cells immediately after isolation as previously described (Schmid *et al.* 2002). RNA concentration was assessed by measuring the absorbance at 260 nm and was adjusted to 1 μg/μL. RNA integrity and absence of genomic DNA were verified by fractionating samples by electrophoresis in denaturing gels. Gels were stained with ethidium bromide and the ratio of 28S to 18S RNA was quantified. Two micrograms of RNA were RT using first strand cDNA synthesis kit according to manufacturer’s instructions (Amersham Biosciences, Buckinghamshire, UK).

### Quantitative real-time PCR analysis

The oligonucleotide primers used are listed in Table S1. Primers were chosen to anneal with a DNA template at a temperature of 60°C and to encompass a coding fragment of 100–300 bp. qPCR was performed as previously detailed in Melchior *et al.* (2002). In brief, a constant amount of 200 ng cDNA from each RT or each dilution of the appropriate standard, was amplified in 25 μL of TaqMan PCR Core Reagent (Applied Biosystems, Carlsbad, CA, USA) according to the manufacturer’s instructions. The reaction mixture consisted of 0.5 U of AmpliTaq Gold polymerase, each of the four dNTPs (0.2 mM), with dUTP replacing dTTP, each pair of primers (300 nM), and MgCl_2_ (3 mM final concentration) in the above-described Tris buffer. Amplifications were performed in an ABI Prism 7700 Sequence Detector System (Applied Biosystems). The reaction was started with a step of 10 min at 50°C for the removal of uracyl residues incorporated into the cDNAs with 1 U of uracyl-*N*-glycosylase (AmpErase reagent). This was followed by an incubation of 10 min at 95°C for AmpliTaq Gold activation, then 40 cycles each consisting of 15 s at 95°C and 1 min at 60°C. At the end of each experiment, amplification products were fractionated by gel electrophoresis to verify that they migrated as a single band with the expected size. Each sample was analyzed in duplicate. For the quantitative analysis, direct detection of PCR products was performed by measuring the progressive increase in fluorescence emitted by the binding of SYBR Green to double-stranded DNA.

Data were normalized to the endogenous reference hypoxanthine phosphoribosyl transferase (HPRT); then using the comparative cycle threshold (CT) method, the amount of RNA transcripts were expressed as a reference to a standardized unstimulated microglia sample. CT was defined as low as transcripts were abundant. For validation of the ddCt method, we also used standards for calibration of HPRT and Trem2 cDNA. Briefly, PCR fragments that served as standards for calibration of quantitative PCR were purified from preparative gels with an extraction kit (QiAquick; Qiagen Germany). Fragment concentrations were assessed by measuring their absorbance at 260 nm and according to their respective molecular weights; these standards were subsequently diluted to obtain 10^7^, 10^6^, 10^5^, 10^4^, 10^3^, and 10^2^ copies per well. The copy number was obtained by relating the fluorescence measured at the CT in unknown samples to the calibration curve derived from the standards. Using the standard curve, it was verified that the absolute copy number of HPRT transcripts was of the same order of magnitude in all samples analyzed by qPCR.

### *In situ* hybridization

*In situ* hybridization was performed on free-floating cryosections sections as previously described (Schmid *et al.* 2002; Thrash *et al.* 2009, Papenfus *et al.* 2007). Briefly, coronal sections were hybridized at 55°C for 16 h with a ^33^P-labeled riboprobe (10^7^ cpm/mL). Myeloid cells and blood vessels were identified by their ability to bind biotinylated tomato lectin (Vector Laboratories, Burlingame, CA, USA). Bound biotinylated tomato lectin was visualized by standard streptavidin, horseradish peroxidase-diaminobenzadine (DAB) methodology. Sections were mounted onto Fisherbrand (Fisher Scientific, Pittsburgh, PA, USA) superfrost/plus slides and dehydrated with ethanol and chloroform. Slides were exposed for 3 days to Kodak X-AR film and dipped in Ilford K-5 emulsion (Polysciences, Warrington, PA, USA). After 3 weeks, slides were developed with Kodak D19 developer, fixed, and counter-stained with Mayer’s hematoxylin. Riboprobes were prepared from antisense sequences corresponding to nucleotides 1282–1768 at the extreme 3′-end of the untranslated region of the large CXCL14 transcript to nucleotides 443–1005 within the open-reading frame of the published CXCL14 sequence (accession #: AF192557) and to the final 600 nucleotides within the 3′ untranslated region of C1qA (accession #: BC002086). Riboprobes for Trem2 were generated from reported plasmids as previously described (Schmid *et al.* 2002).

### Northern blot

For Northern blots, 2 μg per lane of poly A+ or 10 μg per lane total RNA were resolved by electrophoresis in a 1.5% agarose/1.2 M formaldehyde gel, transferred to nylon membrane, and hybridized with ^32^P-radiolabeled probes (Schmid *et al.* 2002 and Thrash *et al.* 2009).

### TOGA and bioinformatic comparison of microglial and macrophage transcriptomes

TOGA (Total Gene expression Analysis) analysis was performed as previously described (Schmid *et al.* 2002) on mRNA samples prepared from microglia and macrophages isolated from LPS/IFNγ-injected murine CNS. Briefly, RNA samples were converted to cDNA using a degenerate pool of biotinylated phasing primers that initiated synthesis at the beginning of the poly(A) tail on each mRNA. The cDNA collection was digested exhaustively with the restriction endonuclease *MspI* and the 3′ fragments were isolated by strepavidin-bead capture. These were released from the beads by cleavage with *Not*I, which recognized a site in the phasing primers. The captured fragments were modified at their 5′ ends to harbor a start site for *in vitro* transcription by ligation of an oligonucleotide adapter to the *MspI* overhang. Following incubation of the modified fragments with T3 polymerase, a collection of RNA fragments was produced corresponding to the 3′ portion of each starting mRNA, from its most 3′*MspI* recognition sequence to the beginning of its poly(A) tail, with each fragment flanked by linker tags of known sequence. For the initial PCR step, first-strand cDNA was prepared from the RNA pool by RT and used in four separate PCRs, in which a 5′ primer that extends by one of four possible nucleotides beyond the *MspI* site (N_1_ position) was paired with a universal 3′ primer to generate an N_1_-specific double-stranded DNA template. In the final step, 256 primers corresponding to all possible permutations of the four nucleotides immediately adjacent to the *MspI* recognition site (N_1_N_2_N_3_N_4_) were matched with the appropriate N_1_ template and utilized in individual robotically performed PCRs paired with a fluorescent primer to produce 256 non-overlapping pools of products, and these products were separated by electrophoresis. This process assigned each product a digital sequence tag address: an eight-nucleotide sequence (the four *MspI* recognition and the adjacent four parsing nucleotides) and a length, both of which were attributes of the individual mRNAs. The amplitudes of the fluorescent PCR products corresponded to the initial concentrations of their parent mRNAs. The amplitudes were automatically collated into a database, indexed by their digital sequence tag addresses that could be queried electronically to identify mRNAs, the concentration of which differed among the experimental samples.

Duplicate TOGA analysis of gene expression was performed on each of the cell population samples described in Fig. 1c and expression of 19023 different mRNAs was detected. Between each of the samples, pair-wise comparisons were made of the relative expression levels of ∼19000 mRNAs detected by TOGA. From these comparisons, linear correlation coefficients were calculated in a pair-wise fashion for each of the samples. The difference index (DI) between each sample was simply calculated as 1 minus the correlation coefficient. The pairs of samples with the highest correlation coefficients (i.e., pairs with the least difference between samples) provided the first branching points. The peak data for these pairs were then averaged and the resulting data set was used to calculate new correlation coefficients with other combined data sets to identify the next branch points. This process was continued until all samples were connected in a complete tree as shown in Fig. 1c.

## Results

Functional differences between CNS-resident microglia and acutely infiltrating macrophages have been identified (reviewed in Carson *et al.* 2006a,b, 2007), but as yet markers able to distinguish these cell types in histological samples have not. We previously compared differences in gene expression between primary neonatal mixed glial culture microglia and peritoneal macrophages treated for 22 h with LPS (100 ng/mL) and IFNγ (10 U/mL) using the gene expression profiling method, TOGA (Schmid *et al.* 2002; Carson *et al.* 2004). Here, we used real-time qPCR and biological replicate samples of neonatal microglia and peritoneal macrophages to validate and quantify the degree of microglial-enriched expression of the 15 TOGA-identified molecules (Table 1, column 1). Enriched expression in neonatal microglia ranged from 2-fold for TCD1D16, Trem2, and Tmem176b (also known as TORID, LR8) to 100-fold for complement component C1qA, GPR84, and hypothetical protein LOC620695. The functions of many of these molecules are still unknown. However, consistent with identified neuroprotective functions of microglia, Trem2 and the tolerance-associated transcript, Tmem176b, each have demonstrated functions in decreasing adaptive immune responses when transfected into peripheral immune cells (Louvet *et al.* 2005; Takahasi *et al.* 2007).

**Table 1:** Comparison of differential gene expression in microglia and macrophages when stimulated in culture versus when stimulated within the intact adult murine CNS by LPS/IFN

Gene	Relative expression *in vitro*: neonatal cultured microglia/peritoneal macrophages	Relative expression *ex vivo*: adult CNS microglia/CNS-infiltrating macrophage
LOC620695	250.00	0.72
GPR84	100.00	0.76
Sal3	100.00	0.56
C1qA	100.00	2.56
NAV3	33.33	0.63
USP2	20.00	0.68
Tem7R/Plxdc2	14.29	1.14
Hspa4l	7.69	0.45
CXCL14	6.67	5.56
Rrbp1	6.67	1.49
Trim47	5.00	0.79
SPARC	4.00	1.47
Tmem176b	2.78	1.10
Trem2	2.44	9.09
TDC1D16	2.04	0.56

Shaded rows indicate molecules showing enriched expression in LPS/IFNγ activated microglia *in vitro* and *in vivo*.

To test whether activated neonatal microglia successfully predicted microglial-enriched gene expression in the adult CNS, we quantified relative expression of the 15 TOGA identified molecules in CNS-resident microglia and CNS-infiltrating macrophages isolated from LPS/IFNγ-injected adult murine CNS. Intracerebral LPS/IFNγ injection caused a rapid, robust and transient, and self-resolving CNS inflammation. Specifically, peripheral macrophages (defined as CD45hi cells) comprised < 1% of the myeloid cells isolated from the unmanipulated healthy adult murine CNS, while all CNS-resident microglia (CD45lo cells) were nearly MHC class II negative (data not shown, Carson *et al.* 1998). By contrast, CD45hi macrophages comprised ∼40% of the cells isolated and nearly all microglia expressed detectable levels of MHC class II, 22 h post-intracerebral LPS/IFNγ injection (data not shown; Carson *et al.* 1998). To provide polarized populations of *ex vivo* microglia and macrophages, only cells from the non-overlapping (boxed) regions of the CD45lo and CD45hi flow cytometry dot plot were collected by fluorescence activated cell sorting for subsequent RNA isolation (Fig. 1a).

Despite the high degree of enriched expression in activated neonatal microglia, only 3 of the 15 original TOGA-identified molecules were enriched in activated adult microglia when compared with acutely infiltrating macrophages isolated from LPS/IFNγ injected CNS: C1qA, Trem2, and CXCL14 (Table 1, column 3). The discrepancy between the *in vitro* and *in vivo* data sets was primarily because of increased expression of these molecules by CNS-infiltrating macrophages. For example, Tmem176b was nearly undetectable in peritoneal macrophages (unstimulated or stimulated), but was strongly induced in CD45hi macrophages infiltrating LPS/IFNγ-injected murine CNS (Fig. 1b). These data suggest that *in vitro* studies overestimated the molecular differences between microglia and macrophages because the peritoneal macrophages were not exposed to the regulatory effects of the CNS micro-environment. By contrast the cultured neonatal microglia isolated from mixed glia cultures were exposed to astrocytes and oligodendrocytes *in vitro*.

To determine the relative similarity of total gene expression between CNS-resident microglia and CNS-infiltrating macrophages, we compared the numbers and levels of total mRNA transcripts detected by TOGA and expressed in common between LPS/IFNγ stimulated microglia and macrophages. We previously reported comparative analysis of the ∼15000 distinct mRNAs detected in TOGA profiling studies of neonatal microglia and peritoneal macrophages (Carson *et al.* 2004). In these *in vitro* studies, LPS/IFNγ-stimulated neonatal microglia showed greater similarity in total gene expression to unstimulated microglia (DI of 0.194) than to either unstimulated or LPS/IFNγ-stimulated peritoneal macrophages (DI between microglia and macrophages = 0.359) (Carson *et al.* 2004). By contrast, comparative analysis of the ∼19000 mRNAs detected in the *ex vivo* TOGA profiling studies unexpectedly revealed that microglia isolated from LPS/IFNγ-injected mice showed greater similarity in total gene expression profiles to CNS-infiltrating macrophages (DI: 0.130) from LPS/IFNγ-injected mice than to microglia isolated from unmanipulated healthy adult murine CNS (Fig. 1c). As a control for this bioinformatic analysis, we compared the gene expression similarities of *ex vivo* lymph node B cells, CD4 T cells, CD8 T cells, and bone marrow-derived cultured dendritic cells profiled in the same TOGA experiment. The gene expression patterns of these cells grouped as predicted from known biology and previously published profiling experiments (Fig. 1c). Specifically, both types of T cells showed greater similarity to each other than to B cells and all lymphocytes displayed greater similarity with each other than with all *ex vivo* myeloid cells (microglia and macrophages) (Fig. 1c).

**Fig. 2:**
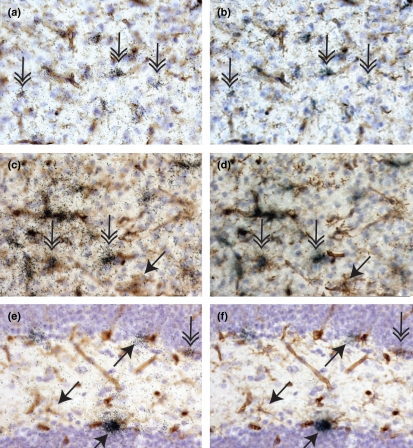
Heterogeneity of microglial gene expression in healthy murine CNS. All panels depict tissue sections prepared from a healthy unmanipulated adult mouse. In all panels, nuclei are labeled in blue with hematoxylin; microglia, macrophages, and blood vessels are labeled with tomato lectin and visualized in brown with DAB. C1qA (panels a, b), Trem2 (panels c, d), and CXCL14 (panels e, f) expression were detected using ^33^P-labeled gene-specific riboprobes. Expression is visualized by induction of black grains in the film emulsion coating each tissue section. In panels a, c, and e, the focal plane is on the level of emulsion. In panels b, d, and f, the focal plane is on level of immunohistochemistry. Downward pointing double arrows indicate examples of microglia positive for gene expression of C1qA (panels a, b), Trem2 (panels c, d), and CXCL14 (panels e, f). Angled downward single arrows indicate examples of microglia negative for gene expression. Upward angled single arrows indicate CXCL14 expression by lectin-negative cells. Panels a–d depict regions within the cortex, panels e and f depict regions within the hippocampus. DAB, diaminobenzadine.

C1qA and Trem2, two of the three molecules identified by qPCR as being enriched in adult microglia isolated from inflamed adult CNS (Table 1), are known to be expressed by microglia (Schmid *et al.* 2002; Takahasi *et al.* 2007; Farber *et al.* 2009; Thrash *et al.* 2009). Microglial expression of both C1qA (Fig. 2a and b) and Trem2 (Fig. 2c and d) was readily detected in both the healthy and inflamed adult CNS using *in situ* hybridization analysis. However, the *in vivo* expression patterns of the two molecules differed in microglia. In Fig. 2, we present the data from the healthy adult CNS, which lacks inflammatory macrophage infiltrates to clearly depict microglial (and not macrophage) expression of these molecules. While nearly all microglia expressed C1qA, the level of expression varied by twofold even in the healthy CNS when the number of emulsion grains per microglia was used as a measure of relative expression (Fig. 2a and b). By contrast, not all microglia expressed Trem2 in the adult CNS. In Fig. 2c and d, Trem2-positive- and Trem2-negative microglia are found in close proximity within the same brain region. These data demonstrate that while expression of these molecules was enriched in adult microglia, neither C1qA nor Trem2 is likely to be a useful universal marker distinguishing microglia from macrophages in CNS tissue sections. These data also demonstrate that microglias are not just heterogeneous between brain regions or during inflammatory responses but even within the same healthy brain region.

**Fig. 3:**
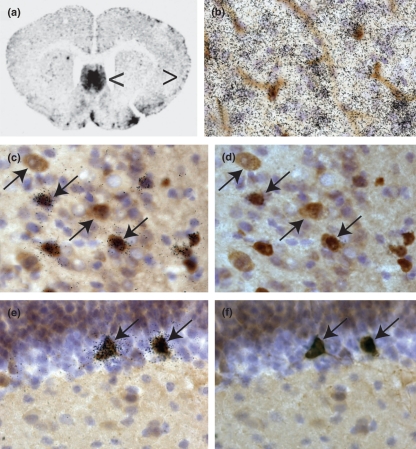
CXCL14 expression in healthy murine CNS in microglia and GABA+ neurons. Panel a is a single autoradiogram from the series depicted in Fig. S1. Left pointing arrow in panel a identifies region of septum depicted in panel b. Right pointing arrow in panel a depicts cortical area depicted in panels c and d. In panels b–f, nuclei are labeled in blue with hematoxylin. In panel b, microglia and blood vessels are labeled with tomato lectin and visualized brown with DAB. In panels c–f, GABA immunoreactivity is visualized in brown with DAB. CXCL14 expression was detected using ^33^P-labeled gene-specific riboprobes. Expression is visualized by induction of black grains in the film emulsion coating each tissue section. In panels b, c, and e, the focal plane is on the level of emulsion. In panels d and e, the focal plane is on level of immunohistochemistry. Downward-angled single arrows indicate examples of CXCL14+, GABA+ neurons in the cortex (panels c, d) and in the hippocampus (panels e, f). The upward pointing arrow indicates an example of CXCL14 negative, GABA+ neurons in the cortex (panels e, f). DAB, diaminobenzadine.

**Fig. 4:**
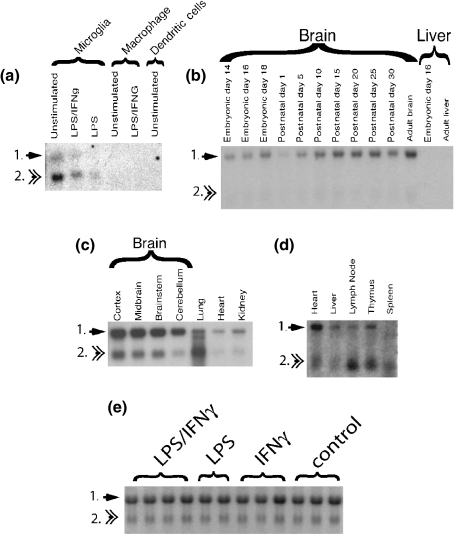
CXCL14 expression in cultured cells and murine tissues. Arrows point out the two known CXCL14 transcripts. (a) CXL14 expression in cultured cells. Cells were stimulated for 22 h with LPS (100 ng), IFNγ (10 U/mL) or LPS + IFNγ (10 U/mL, 100 ng/mL). (b) Developmental expression of CXCL14 in the CNS and liver. (c and d) Tissue distribution of CXCL14 expression. Northern blot in c was exposed overnight. The Northern blot in d was exposed for 2 weeks; e) each lane represents RNA isolated from a single mouse CNS that was unmanipulated or which received 5 μL intracerebral injections of IFNγ (10 U/mL), LPS (100 ng/mL) or IFNγ + LPS (10 U/mL, 100 ng/mL) 22 h prior to isolation.

CXCL14 was the third molecule identified as microglial-enriched from the qPCR screen and was also a demonstrated chemoattractant for B cells and monocytes. CXCL14 has been previously detected in CNS RNA but the brain regions and cells expressing CXCL14 had not been reported (Hromas *et al.* 1999; Cao *et al.* 2000; Frederick *et al.* 2000; Vadasz *et al.* 2007; Meuter and Moser 2008). Here, we examined the expression pattern of CXCL14 in unmanipulated healthy and LPS/IFNγ-injected adult murine CNS using two non-overlapping riboprobes that showed identical labeling in tissue sections (Figs. 2, 3 and Fig S1). CXCL14 expression was not regulated *in vivo* by LPS/IFNγ (Fig. 4e, and *in situ* hybridization data not shown). Therefore, CXCL14 expression is depicted only by the unmanipulated healthy adult CNS in Figs. 2e and f and 3 and Fig. S1. Surprisingly, the majority of CXCL14 expression was detected on lectin-negative cells (upward pointing arrows Fig. 2e and f). While most microglia were negative for CXCL14 expression (downward pointing single arrows Fig. 2e and f), low-level expression of CXCL14 was detected in rare microglia within the hippocampus, (downward pointing double arrow Fig. 2e and f) and in microglia located in the septum (Fig. 3a and b) and lateral striatum (Fig. S1 and data not shown). Within these last two regions, all cells including microglia expressed high levels of CXCL14 (Fig. 3a and b, Fig. S1). These data indicate that while CXCL14 mRNA is enriched in RNA pooled from all adult *ex vivo* microglia, CXCL14 expression is characteristic of only a very small subset of microglia *in vivo*.

An overview of CXCL14 expression in the entire brain is depicted and described in Fig. S1. In brief, the highest levels of CXCL14 expression were observed in the septum, the islands of Calleja, the interpeduncular nucleus, the hippocampus, and the Purkinje layer of the cerebellum (Fig. S1). Cells expressing high levels of CXCL14 were found throughout the cerebral cortex but were most abundant in the cingulate cortex. In the striatum, CXCL14 expression delineated a lateral region of the striatum that has not been previously recognized as a defined striatal structure. Most CXCL14+ cells were identified as glial fibrillary acidic protein negative, 2′3′-cyclic nucleotide phosphodiesterase P-negative, and NeuN positive (data not shown). While nearly all CXCL14-positive cells displayed a GABAnergic neuronal phenotype (GABA-positive, large nucleus, and cell body: downward pointing arrows, Fig. 3b–d), not all GABAnergic neurons were CXCL14 positive (upward pointing arrows, Fig. 3b and c).

Finally, we examined CXCL14 expression *in vitro* and *in vivo* by Northern blot analysis (Fig. 4). CXCL14 is encoded by two mRNA transcripts with identical open reading frames (Hromas *et al.* 1999; Cao *et al.* 2000; Frederick *et al.* 2000; Vadasz *et al.* 2007; Meuter and Moser 2008). In cultured microglia (Fig. 4a), peritoneal macrophages (Fig. 4a), thymus (Fig. 4d), and secondary lymphoid structures (Fig. 4d; lymph node, spleen) the smaller CXCL14 transcript predominates (transcript 2, Fig. 4). By contrast, in non-immune organs including the CNS, the larger transcript predominates (transcript 1, Fig. 4a, c and e). Northern blot analysis confirmed enriched CXCL14 expression in cultured neonatal microglia when compared with peritoneal macrophages or bone marrow-derived dendritic cells (Fig. 4a). Because primary microglia from mixed glial cultures were derived from neonatal CNS cortices, we examined whether microglial expression of CXCL14 was more abundant in the embryonic or early postnatal CNS. By Northern blot analysis, the ratio of larger to smaller CXCL14 transcript did not change during development (Fig. 4b). By *in situ* hybridization analysis, the identical pattern of CXCL14 expression was observed at all ages examined from postnatal day 1 to 3 months (data not shown and Figs. 2 and 3, Fig. S1). These data indicated that detection of high levels of CXCL14 expression in neonatal microglia from mixed glial cultures was not a consequence of these cells reflecting an earlier stage of CNS development. Rather the robust expression of CXCL14 in cultured neonatal microglia was probably an artifact of culture in the absence of signals provided by an intact functional CNS.

## Discussion

Several studies have identified functional differences in CNS-resident microglia and acutely infiltrating inflammatory macrophages (reviewed in Carson *et al.* 2007). However, as yet a universal marker that could be used to distinguish these functionally different cells in inflamed CNS tissue sections has not been identified. Here, we tested and confirmed by qPCR that a panel of 15 molecules previously identified by TOGA gene profiling were enriched in LPS/IFNγ-stimulated neonatal microglia when compared with similarly treated peritoneal macrophages.

Despite enriched expression from 2-fold to 250-fold in activated neonatal microglia *in vitro*, the majority of these TOGA-identified were not preferentially expressed in CNS-resident microglia when compared with CNS-infiltrating macrophages isolated from LPS/IFNγ-injected adult murine CNS. In large part, the failure to confirm preferential expression of 12 molecules in LPS/IFNγ-activated CNS-resident microglia was because of much higher expression of these molecules in CNS infiltrating macrophages (CD45hi cells) when compared with peritoneal macrophages (also CD45hi cells). Three molecules were confirmed by qPCR as enriched in adult microglia from LPS/IFNγ-injected adult CNS (C1qA, Trem2, CXCL14), but *in situ* hybridization analysis demonstrated that expression of these three molecules was heterogeneous in adult microglia in both the unmanipulated and inflamed CNS. Developmental regulation accounts for the observed heterogeneous pattern of microglia gene expression only in part. Before postnatal day 7, all microglia were Trem2-positive (Thrash *et al.* 2009). After eye opening in the pup, only subsets of microglia expressed varying levels of Trem2 (Schmid *et al.* 2002; Carson *et al.* 2006a,b; Thrash *et al.* 2009). By contrast, microglial expression of CXCL14 did not vary from postnatal day 1 adulthood.

The significance of CXL14 expression in the intact functional CNS is unknown. Because CXCL14 is a macrophage chemoattractant, it is tempting to speculate that it may contribute to organizing the close apposition of microglia to GABA-positive, CXCL14-positive neurons. However, as yet, microglia have not been formally demonstrated to respond to CXCL14 nor has the receptor for CXCL14 been identified. In addition, it is unknown why regions with high levels of CXCL14 expression do not show increased susceptibility to macrophage or B-cell infiltration. Is this an evidence of active immuno-suppression by the CNS environment or evidence of the failure to produce active bioavailable CXCL14 protein?

In aggregate, these data contribute to the growing literature demonstrating that microglia cultured in the absence of signals provided by an intact functional CNS do not fully mimic microglia located within the intact adult CNS (reviewed in Carson *et al.* 2007). Here, we show that cultured microglia expressed high levels of molecules such as CXCL14 that were rarely expressed by these cells *in vivo*. In addition, these data confirmed that microglial gene expression was not homogeneous throughout the CNS. Indeed, microglial phenotype, both *in vivo* and *in vitro* appeared to be determined by local environmental cues. Furthermore, the continuing failure to identify a definitive microglial-specific marker suggests that any molecule expressed by all microglia subsets in all micro-environments will also be expressed by nearly all inflammatory macrophages. Finally, induced expression of ‘microglial-specific’ molecules with immunosuppressive functions by CNS-infiltrating macrophages adds to the growing literature that the CNS actively regulates microglia and macrophage phenotypes via a wide array of neuronal and glial-expressed molecules (including CD22, CD200, fractalkine, and vasoactive intestinal protein) (Getts *et al.* 2008 and reviewed in Carson *et al.* 2007). The existence of active regulation of CNS inflammation by the CNS itself has consequences for the aging and diseased CNS. Failures in CNS immunoregulation have the potential to contribute to the onset and progression of chronic neuroinflammation characteristic of many neurodegenerative disorders.

## Abbreviations used

CT: cycle threshold
DI: difference index
HPRT: hypoxanthine phosphoribosyl transferase
IFN: interferon
LPS: lipopolysaccharide
MHC: major histocompatability complex
TOGA: Total Gene expression Analysis

## Supporting Information

**Table S1** qPCR primer pairs.

**Figure S1** CXCL14 expression in unmanipulated adult murine CNS.
