# Supplementary material for: Differential gene expression in LPS/IFNγ activated microglia and macrophages: in vitro versus in vivo
Source: J Neurochem. 2009 May;109(Suppl. 1):117–25. doi: 10.1111/j.1471-4159.2009.05984.x (PMC2766614; doi:10.1111/j.1471-4159.2009.05984.x)
Supplement: Supplementary file 1 [file jnc0109-0117-SD1.doc]

Supplemental table 1: qPCR primer pairs

| ***Gene*** | ***Accession #*** | ***Primer 1 (forward)*** | ***Primer 2 (reverse)*** |
| --- | --- | --- | --- |
| LOC  620695 | XM_885087 | CTTTCGCTGAACACTTTTTATAATTG | CTCACAGACAACATCCAGGTAAAA |
| GPR84 | NM_030720 | ATCCCCTTCCTGTTGCTCAA | CTGTGAAATACCCAAGGACTGAA |
| Sal3 | AC100120 | AGCCACTTCTCCCACACCAT | TCGAGCCGCCACTATTCA |
| C1qA | BC002086 | CACACGGGTCGCTTCATCT | GAAAATGAGGAATCCGCTGAA |
| NAV3 | AC100120 | AGCCACTTCTCCCACACCAT | TCGAGCCGCCACTATTCA |
| USP2 | AK047204 | CCCAAGCATCGTCACAGTAGA | AACATGGCACGGGGATAAA |
| Tem7R/  Plxdc2 | AF378761 | ACTTGCCAGGAGTGTGACGA | TTGTCATATTTTTTTTCCTTGATTTC |
| Hspa4l | NM_011020 | CTGCGACACTAATGCTAAGCCT | ACAAAAATCAATTAAGAAAACCAACA |
| CXCL14 | NM_019568 | ATAAGGGGTTTTGTATTTGTCCAT | CATGCTCACTGTTCCTCCCA |
| Rrbp1 | BC031452 | TGGATATCTACGACACTCAGACCTT | CTTCTTCATACGACGTCTCCTTC |
| Trim47 | NM_172570 | ACTAGCCTCAGAGACTCCCACAC | AGTTGTCCCAGCTAAAGATCTGTT |
| SPARC | NM_012656 | CTCAAGAGGAAGACAGAAAAGGTT | TGAAATGCTTGGAGGGGAA |
| Tmem176b | AK007408 | GTGTCCTGTCCATAGTAATCCA |  |
| Trem2 | NM_031254 | ACAGCACCTCCAGGAATCAAG | CCACAGCCCAGAGGATGC |
| TDC1D16 | BC026530 | CTGAAGAAAAATGAGCCACAAAG | AACAGAGAAACGAATCACTTGCTAT |
| HPRT | BC004686 | AGATGCTGTTACTGATAGGAAATTGA | CCCTCTGGTAGATTGTCGCTTA |
