# Supplementary figures and images for: Differential gene expression in LPS/IFNγ activated microglia and macrophages: in vitro versus in vivo
Source: J Neurochem. 2009 May;109(Suppl. 1):117–25. doi: 10.1111/j.1471-4159.2009.05984.x (PMC2766614; doi:10.1111/j.1471-4159.2009.05984.x)

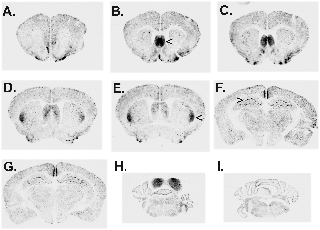

Supplement: Supplementary file 2 [file jnc0109-0117-SD2.tif]
